# The Management of Drug-Induced Gingival Enlargement in a Patient With Preexisting Periodontitis

**DOI:** 10.7759/cureus.52190

**Published:** 2024-01-12

**Authors:** Lekha Alanija, Raaja Sreepathy Chandran Selvaraj, Shanmugapriya Ramamurthy, Arunmozhi Ulaganathan, Vikram C

**Affiliations:** 1 Periodontology, Sri Venkateswara Dental College and Hospital, The Tamil Nadu Dr. M.G.R. Medical University, Chennai, IND

**Keywords:** drug-influenced gingival enlargement, amlodipine, gingival overgrowth, calcium channel blockers, periodontal therapy

## Abstract

Antihypertensives such as amlodipine, which is a family of calcium channel blockers (CCBs), possess a limitation by causing gingival enlargement on long-term use. Gingival enlargement hinders the patient's oral hygiene maintenance and causes more plaque accumulation and inflammation. The severity of the condition is dependent on dose and duration. When untreated, this leads to functional and esthetic disabilities. This is a case report of amlodipine-induced gingival enlargement in a young, chronic periodontitis patient who was under 5 mg of amlodipine for six months. Upon diagnosis, the patient underwent periodontal surgery and supportive periodontal therapy, which significantly improved her periodontal health in a one-year follow-up period.

## Introduction

Gingival enlargement is one of the clinical signs of gingival inflammation. It is a proliferative condition caused by an increase in fibroblast number and collagen fibers and a decrement in collagenolysis. Collagenase, which is a type of matrix metalloproteinase (MMP), fails to get activated due to reduced folate uptake in fibroblast. This results in the decreased degradation and remodelling of the connective tissue matrix leading to gingival enlargements [1]. The varied etiological factors are plaque accumulation on the tooth surface, infection, the presence of foreign bodies, hereditary, systemic diseases, and also the influence of certain systemic drugs. This condition causes difficulty in speech, mastication, and unpleasant esthetics [2]. The enlargement of the gingiva, which is caused by the influence of drugs, is called drug-induced gingival hyperplasia or drug-induced gingival overgrowth (DIGO). There are a wide array of drugs that cause gingival overgrowth, the most common of them are anticonvulsants, calcium channel blockers (CCBs), and immunosuppressants [3].

CCBs such as nifedipine and amlodipine, which are dihydropyridine derivatives, are most commonly used for the treatment of hypertension. CCBs block the intracellular influx of calcium, resulting in the dilatation of peripheral vasculature and thereby reducing hypertension [4]. Amlodipine-induced gingival enlargement appears to be dose- and duration-dependent. This is a case report of amlodipine-induced gingival overgrowth within a short period of time in a young patient with preexisting periodontitis.

## Case presentation

**Figure 1:**
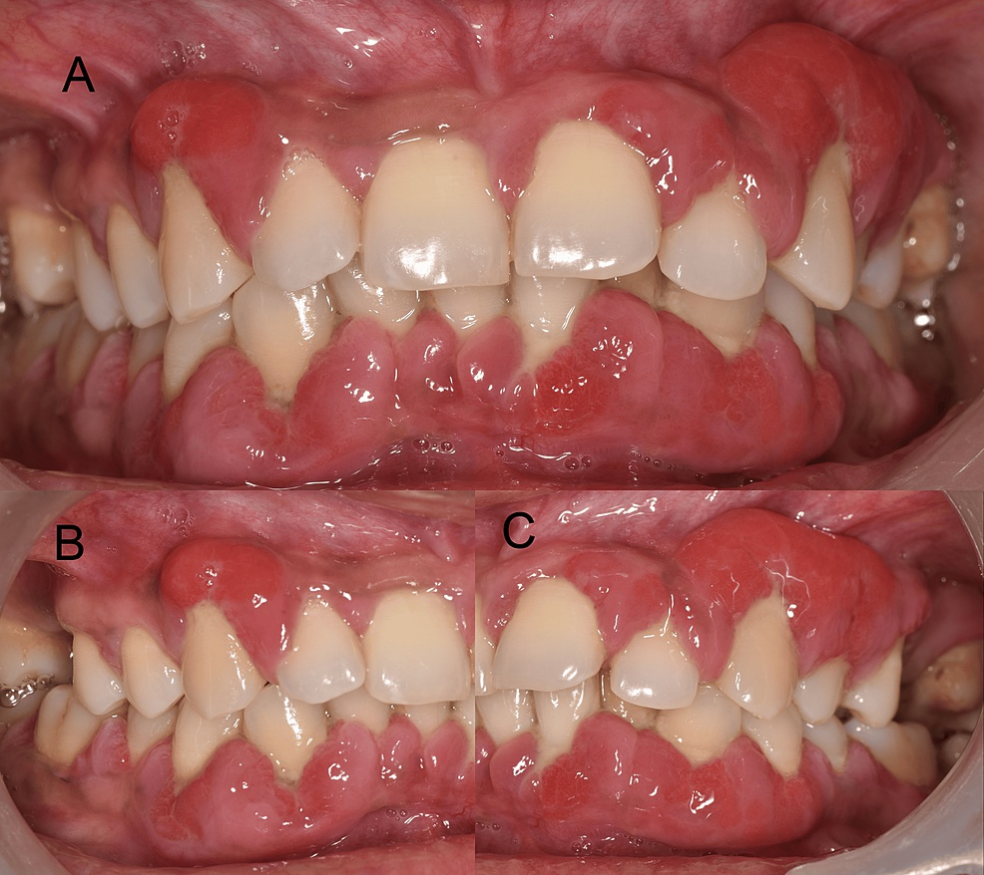
Preoperative buccal view A, B, and C depict the overall preoperative gingival enlargement with signs of acute inflammation in reference to maxillary and mandibular anteriors

A 34-year-old female patient reported gingival swelling that appeared initially in the maxillary right canine region and then progressed to reach the current state, covering the entire of her maxillary and mandibular teeth. The patient had undergone uneventful molar teeth removal five years before, due to advanced caries and attachment loss (Figure 1).

**Figure 2:**
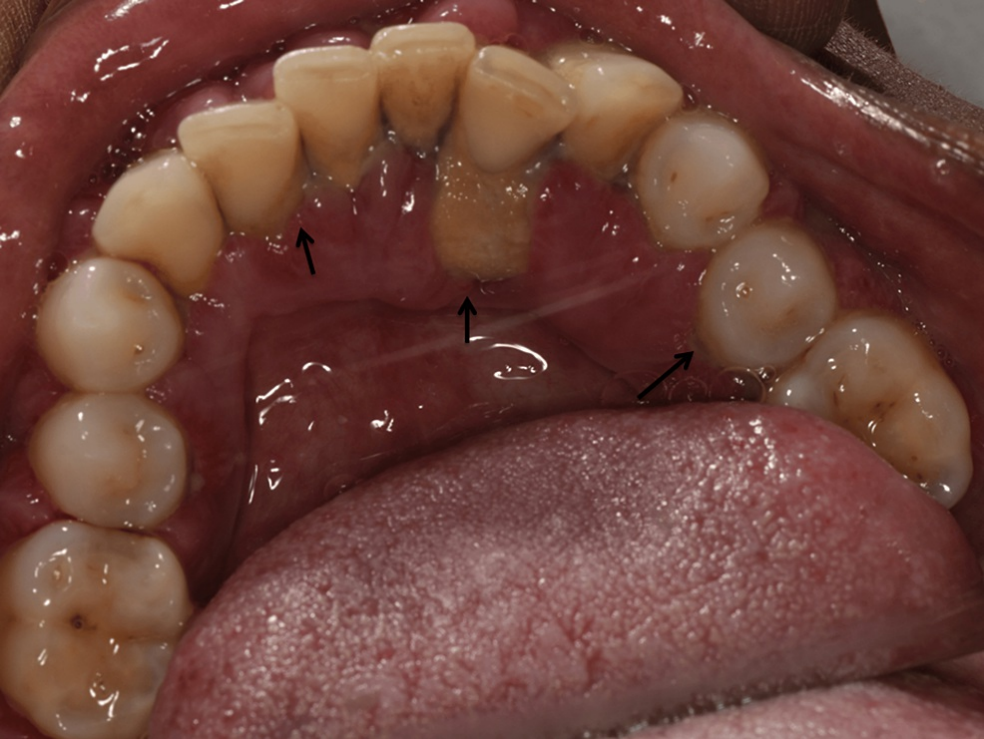
Preoperative lingual view Gingival enlargement with deposits of calculus

It bled profusely while brushing and caused discomfort during mastication. The patient was a known hypertensive under amlodipine 5 mg, twice a day for the last six months, and the gingival swelling was observed for three months. On examination, a generalized moderate amount of supra- and subgingival calculus was noted (Figure 2).

**Figure 3:**
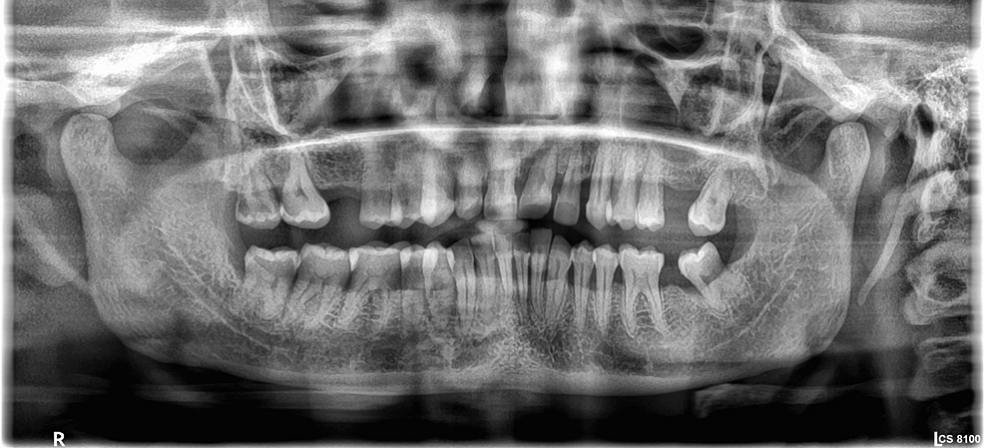
Orthopantomograph Generalized horizontal bone loss until the middle third of the roots as observed in the orthopantomograph

According to the Simplified Oral Hygiene Index (OHI-S) by John C. Greene and Jack R. Vermillion (1964), the patient had a poor oral hygiene score of 4.3. There was a diffuse gingival enlargement of both arches with signs of acute inflammation in reference to the anteriors. Generalized periodontal pockets with an average attachment loss of 5-6 mm were observed. Considerable tooth mobility of grade II (Miller's classification, 1950) was noted in 14, 15, 24, 25, 31, and 41. And the rest of the majority of the teeth exhibited mild grade I mobility. There was a grade II furcation involvement (Glickman's classification, 1953) in 17 and 27 and grade I in 36 and 46. On radiographic investigation, generalized horizontal bone loss extending approximately until the middle third of the roots was seen (Figure 3).

**Figure 4:**
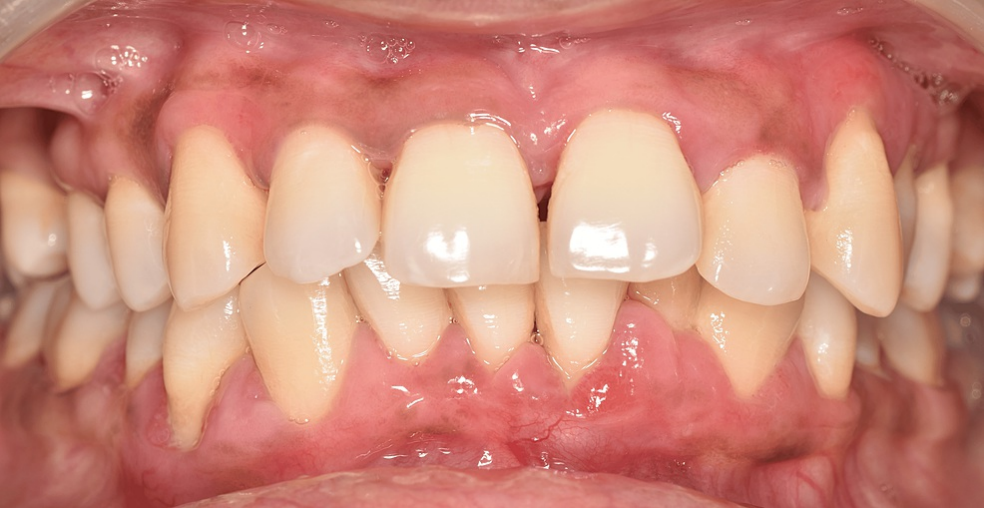
After phase I therapy of scaling and root planing Partial resolution of gingival inflammation and enlargement after repeated episodes of nonsurgical therapy

The patient's hemogram reports were within normal limits and so her blood glucose measurements. Based on the average attachment loss of >5 mm and grade II furcation involvement in the maxillary molars with an estimated percentage of bone loss/age of 2.2, the patient was diagnosed as stage III, grade C periodontitis with drug-induced gingival overgrowth. So, a comprehensive periodontal management was planned to address both the periodontal inflammation and drug-induced gingival enlargement. Initially, phase I therapy including scaling and root planing was done after the substitution of the drug (amlodipine 5 mg) by telmisartan 40 mg. There was a substantial reduction in the size of the enlargement after repeated scaling and root planing in a time period of three weeks. But deep pockets of 7-9 mm persisted in relation to the maxillary second quadrant and mandibular anteriors. In mandibular posteriors and maxillary first quadrant, the pocket depth was around 6 mm. Therefore, open flap debridement was planned from 14-24 to 36-48. Following medical fitness, after patient reassurance, surgery was initiated. On the day of the surgery, blood pressure was assessed priorly, and the procedure was done under 2% lignocaine with 1:200000 adrenalin. The access flap was raised, and thorough debridement was done. After flap approximation, the patient was reassessed for bleeding and then dismissed. Postoperatively, the patient was put under analgesics and antibiotics for five days and was instructed to avoid eating chewy food on the operated site. The patient was instructed not to brush on the operated site and instead was asked to use chlorhexidine 0.02% mouth rinse. All the four quadrants were operated with a one-week interval between them (Figure 4).

**Figure 5:**
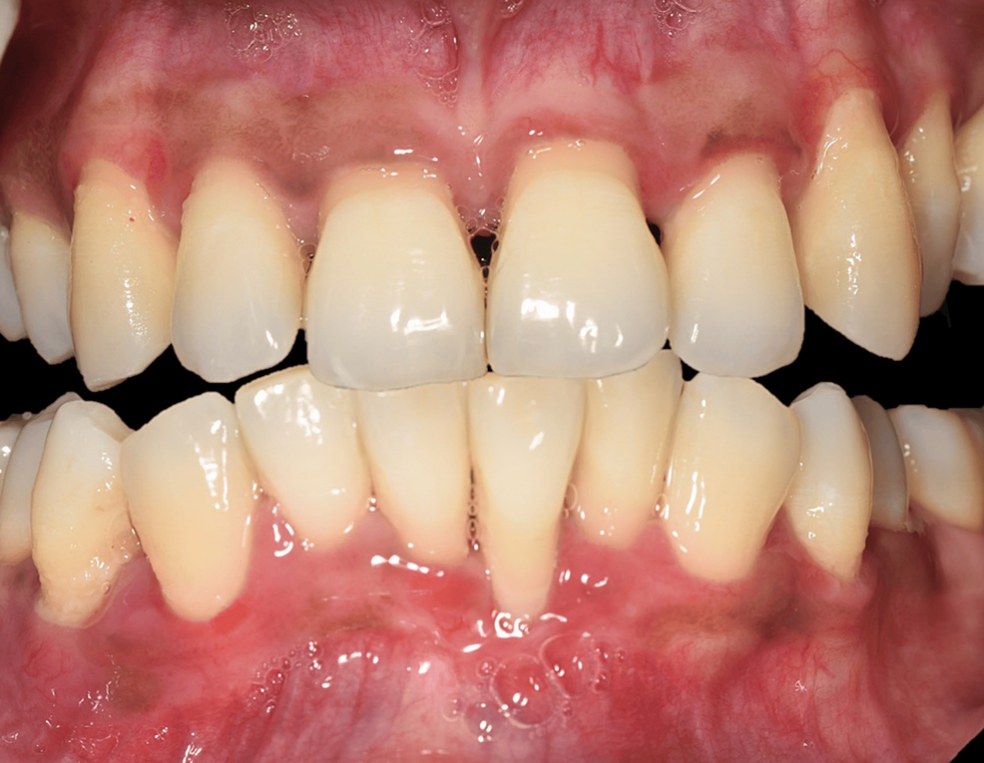
Reevaluation of surgical phase Postoperative healing as observed after one month of periodontal surgical therapy

Postoperative healing after one month was appreciable with an average pocket depth reduction of 2-4 mm (Figure 5).

**Figure 6:**
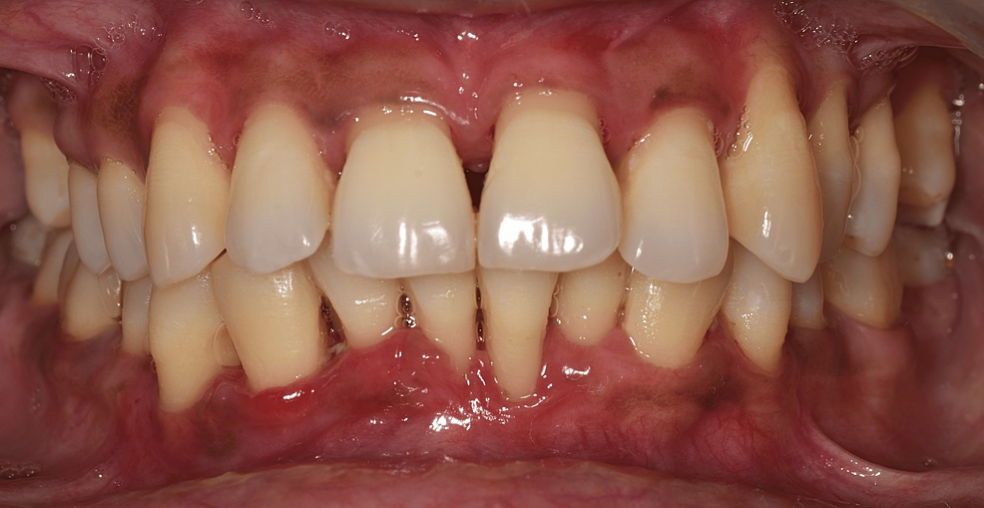
Review of the periodontal maintenance at one-year follow-up

The patient was under supportive periodontal care, and a recent one-year follow-up exhibited no relapse of gingival enlargement with adequate periodontal health maintenance (Figure 6).

## Discussion

Calcium channel blockers are one of the first-line drugs used in the treatment of hypertension [5]. Among this group, amlodipine is a commonly prescribed drug (around 79.6%) [6]. On long-term use, gingival overgrowth was reported as one of its side effects. Drug-induced gingival overgrowth is influenced by a number of risk factors. The non-modifiable risk factors are age and genetic predisposition, and the modifiable risk factors are the anatomy of tooth structure, poor oral hygiene, pharmacokinetic variables, periodontal disease, probing pocket depth, and the duration and dose of a drug, and the action of those drugs on growth factors influence the disease pattern [7]. Normally, a lower dose of amlodipine, which is 5 mg or less, results in a longer duration of gingival enlargement development, as shown in the literature [8]. However, in the present case report, the same low dose had resulted in overgrowth in a short period of six months. This rapid presentation could be due to the patient's young age and the presence of periodontitis as a risk factor. Generally, amlodipine induces overgrowth by noninflammatory pathways and inflammatory pathways [9]. In the noninflammatory pathway, the drug causes reduced folic acid absorption, which decreases the collagenase activity. Additionally, there is also the inhibition of aldosterone synthesis by the adrenal cortex that coherently increases the level of adrenocorticotropic hormone causing the upregulation of keratinocyte growth factor [10]. However, in the inflammatory pathway, the accumulation of bacterial plaques because of inadequate oral hygiene and the concentration of the medication in the gingival crevicular fluid have a direct effect in causing inflammation because of the release of pro-inflammatory cytokines and a decrease in collagenase activity [11].

Despite these mechanisms, genetically, there is an existence of variable proportions of fibroblast subsets in each individual. It has been demonstrated that gingival fibroblasts exhibit functional variability in response to a variety of stimuli such as the influence of drugs, lending credence to this notion [12].

To initiate the enlargement, a baseline concentration of drugs in the gingival tissues is required, which is also influenced by the presence of plaque accumulation. Addy et al. (1983) found a positive association between the oral hygiene status of the individual and the prevalence and severity of gingival enlargement [13]. Therefore, the maintenance of stringent plaque control and oral hygiene along with the elimination of causative drug and the replacement with an alternate drug plays an important role in the management of drug-induced gingival enlargements.

In this case report, amlodipine-induced gingival enlargement, which has occurred in a young individual within a short period of the consumption of the drug, had been successfully diagnosed and managed with periodontal therapy with a postoperative follow-up of one year.

## Conclusions

Antihypertensive drugs such as calcium channel blockers have been extensively prescribed. The prevalence of its associated common side effect of gingival enlargement is strongly influenced by the presence of oral microbiota. Early detection and emphasis on regular dental maintenance visits can have an enormous effect on the reduction of the occurrence of drug-induced gingival overgrowth in hypertensive patients.

## References

[1] Seymour RA, Thomason JM, Ellis JS (1996). The pathogenesis of drug-induced gingival overgrowth. J Clin Periodontol.

[2] Camargo PM, Melnick PR, Pirih FQ, Lagos R, Takei HH (2001). Treatment of drug-induced gingival enlargement: aesthetic and functional considerations. Periodontol 2000.

[3] Butler RT, Kalkwarf KL, Kaldahl WB (1987). Drug-induced gingival hyperplasia: phenytoin, cyclosporine, and nifedipine. J Am Dent Assoc.

[4] Tonsekar P, Tonsekar V (2021). Calcium-channel-blocker-influenced gingival enlargement: a conundrum demystified. Oral.

[5] Fares H, DiNicolantonio JJ, O'Keefe JH, Lavie CJ (2016). Amlodipine in hypertension: a first-line agent with efficacy for improving blood pressure and patient outcomes. Open Heart.

[6] Savage RD, Visentin JD, Bronskill SE (2020). Evaluation of a common prescribing cascade of calcium channel blockers and diuretics in older adults with hypertension. JAMA Intern Med.

[7] Jose JI, Santhosh YL, Naveen MR, Kumar VI (2011). Case report of amlodipine induced gingival hyperplasia - late onset at a low dose. Asian J Pharm Clin Res.

[8] Chaturvedi R, Jain A (2011). Amlodepine induced gingival enlargement - presentation of a clinical case series. J Clin Exp Dent.

[9] Joshi S, Bansal S (2013). A rare case report of amlodipine-induced gingival enlargement and review of its pathogenesis. Case Rep Dent.

[10] Das SJ, Olsen I (2000). Keratinocyte growth factor is upregulated by the hyperplasia-inducing drug nifedipine. Cytokine.

[11] van der Vleuten CJ, Trijbels-Smeulders MA, van de Kerkhof PC (1999). Telangiectasia and gingival hyperplasia as side-effects of amlodipine (Norvasc) in a 3-year-old girl. Acta Derm Venereol.

[12] (2004). Informational paper: drug-associated gingival enlargement. J Periodontol.

[13] Addy V, McElnay JC, Eyre DG, Campbell N, D'Arcy PF (1983). Risk factors in phenytoin-induced gingival hyperplasia. J Periodontol.

